# A Novel Interannual Rainfall Runoff Equation Derived from Ol’Dekop’s Model Using Artificial Neural Networks

**DOI:** 10.3390/s22124349

**Published:** 2022-06-08

**Authors:** Omar Mimeche, Amir Aieb, Antonio Liotta, Khodir Madani

**Affiliations:** 1 Research Laboratory in Applied Hydraulics and Environment (LRHAE), Department of Hydraulics, Faculty of Technology, University of Bejaia, Targa Ouzemour, Bejaia 06000, Algeria; omar.mimeche@univ-bejaia.dz; 2 Laboratory of Biomathematics, Biophysics, Biochemistry, and Scientometric (BBBS), Bejaia University, Bejaia 06000, Algeria; amir18informatique@gmail.com (A.A.); madani28dz2002@yahoo.fr (K.M.); 3 Faculty of Computer Science, Free University of Bozen-Bolzano, 39100 Bolzano, Italy; 4 Research Center of Agro-Food Technologies (CRTAA), Bejaia 06000, Algeria

**Keywords:** rainfall-runoff modeling, water balance model, ANN model, watercourse, De Martonne index, inter-annual time scale, northern Algeria, watershed

## Abstract

In water resources management, modeling water balance factors is necessary to control dams, agriculture, irrigation, and also to provide water supply for drinking and industries. Generally, conceptual and physical models present challenges to find more hydro-climatic parameters, which show good performance in the assessment of runoff in different climatic regions. Accordingly, a dynamic and reliable model is proposed to estimate inter-annual rainfall-runoff in five climatic regions of northern Algeria. This is a new improvement of Ol’Dekop’s equation, which models the residual values obtained between real and predicted data using artificial neuron networks (ANN_s_), namely by ANN_1_ and ANN_2_ sub-models. In this work, a set of climatic and geographical variables, obtained from 16 basins, which are inter-annual rainfall (IAR), watershed area (S), and watercourse (WC), were used as input data in the first model. Further, the ANN_1_ output results and De Martonne index (I) were classified, and were then processed by ANN_2_ to further increase reliability, and make the model more dynamic and unaffected by the climatic characteristic of the area. The final model proved the best performance in the entire region compared to a set of parametric and non-parametric water balance models used in this study, where the R^2^_Adj_ obtained from each test gave values between 0.9103 and 0.9923.

## 1. Introduction

Precipitations are the origin of water resources, which undergo different quantitative and qualitative transformations on the slopes. The losses of rainwater are almost observed as a form of infiltration, retention in the soil, and evaporation. Some part of this quantity can also flow into wadis until it moves into the sea. The estimation of actual evapotranspiration is the component most required for estimating water and energy balance equations [1,2]. This resource can be accessed on the inter-annual scale, according to the history of climatic and hydrometric measurements that could be given in the outlet of some watersheds. For ungauged watersheds, the lack of some information posed a big problem in the estimation of the mean annual flow, which is the main scientific challenge for many hydrologists [3,4,5]. The water balance concept was considered to study the hydrological behavior of watersheds and to describe the relationship between water and thermal components of the earth. This relativity was defined by a mathematical ratio between rainfall (R), rainfall-runoff (RR), and real evapotranspiration (Ea) [6]. The estimation of rainfall-runoff is necessary as the first step to search for the best evaluation of Ea. In literature, the first attempts were started by Schreiber [7] and Ol’Dekop [8]. Then, Budyko [9] proposed an average model of both previous equations to minimize the estimation errors that were given by Schreiber and Ol’Dekop. On the other hand, Pike [10] also gave a new formula to estimate Ea, in the form of a power equation. However, in 1974, Budyko and Miller developed a new theory, which describes the main factors to determine the actual rate of evapotranspiration on the inter-annual scale [6]. This work sparked the interest to understand the effect of watershed climate characteristics, vegetation, and capacity of watershed storage on Ea estimation [11,12,13]. In this regard, several conceptual models cited in the literature show the best performance when using physical indices (Soil, vegetation, and the capacity of basin storage) as input variables to assess Ea on daily and intra-daily scales, including black-box models (NARX-NN), machine learning models (ANN, random forest), the GR6J-WGANN2 model, etc. [14,15,16]. On the other hand, climatic variability also impacts the choice and the performance of runoff models applied on the annual and interannual scale, in which different models were developed regionally according to the rainfall characteristics of basins [17,18]. The proposed technique by Budyko was proven to be an effective tool to explain the interactions between a set of climatic factors and the characteristics of watersheds, and also their impacts on the water balance energy; however, its limit of application has always posed a problem for ungauged watersheds [5,19,20]. Furthermore, climatic variability and the aridity ratio of catchment areas also posed a problem for hydrologists trying to propose a dynamic and reliable model for estimating a rainfall-runoff in different regions of the world. Du et al. explained that the Budyko hypothesis was generally used to estimate the rainfall-runoff in arid regions [21]. Moreover, Wu et al. proved that applying Budyko’s model to different climates and watersheds of China, using different time scales, performs well at a mean annual scale where the climate area is arid, which is better than wet areas [22]. Xiong and Guo [23], deduced that Budyko’s non-parametric model, despite being easier for application, has never given good results in the context of long-term water balance studies in humid watersheds. Contrariwise, the parametric models are more performant in humid regions with local optimization of those input parameters. Thus, we can understand that no single model is perfect for an easy application in estimating inter-annual rainfall-runoff, especially in areas characterized by climatic diversity.

This work aims to propose a dynamic model that will be used to estimate inter-annual rainfall-runoff based on modeling residual data distribution (IARR’) given by Ol’Dekop’s model [8] in different areas. The new equation form was obtained by applying an artificial neural network (ANN) on several climatic and geographical input variables, which proved a good correlation with IARR’, such as inter-annual rainfall (IAR), De Martone index (I), watershed area (S), and watercourse (WC), respectively. Furthermore, the model was applied to five climatic floors of northern Algeria according to the climatic and geophysical diversity that characterized this area, which helped to determine the parameters of the new model. Generally, the article is structured in five sections, where a presentation of the study area, data, models, and metrics of comparison are given in the Section 2. Then, the statistical analysis, the bioclimatic classification, the ANNs design, and modelling steps are shown in Section 3. On the other hand, the comparison and the performance analysis with a set of parametric and nonparametric models that are mostly applied to assess IARR are given in Section 4. Finally, the conclusions were drawn in Section 5.

## 2. Material and Methods

### 2.1. Study Area and Data

**Figure 1:**
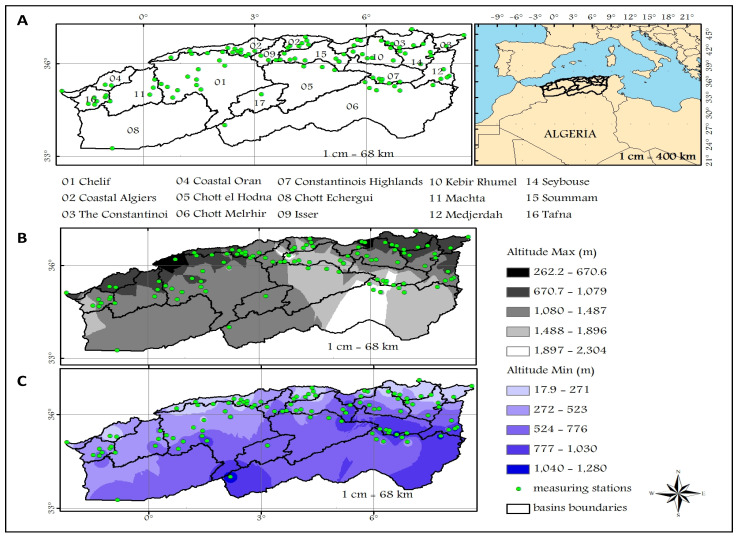
Maps of the northern Algeria region (**A**) and elevation variation (**B**,**C**) of 16 watersheds, showing the hydrometric stations’ locations.

Algeria is an important country in Northern Africa, which is located on the southern shore of the Mediterranean Sea. It is bordered on the east by Tunisia and Libya, in the west by Morocco, and in the southwest by Mauritania and Western Sahara; moreover, in the southern part, it overlooks Niger and Mali. It extends over an area of 2,381,741 km^2^, where 85% of the surface represents the desert region. Our study relates to the northern Algeria area, which includes a surface of 480,000 km^2^ [24]. It is bordered in the north by the Mediterranean Sea, while the southern part overlooks the Great Sahara. It is located between a longitude of −2.2° and 8.6°, and a latitude of 33° and 37°, which is distributed over 16 watersheds numbered from 01 to 17, except basin 13 which represents the southern part of the country (Figure 1A). In this region, the spatial precipitation distribution is very heterogeneous, characterized by a strong gradient from north to south, and a weak one from east to west [25]. North Algeria has a typically Mediterranean climate, which is hot and dry in summer. Contrariwise, in winter, it is mild and rainy. Annual precipitation data varies between 400 mm and 1500 mm. In the highlands and the Saharan Atlas the climate is generally semi-arid, and the rainfall does not exceed 500 mm annually. The pre-Saharan and Saharan regions have a very arid climate, which is almost devoid of precipitation, and the mean annual rainfall there varies between 50 mm and 200 mm [26,27]. The temperature of the country varies between day and night, as well as between summer and winter [28]. According to the data, the mean daily temperature is observed in January with a value between around 10 °C and 12 °C. In July it is comprised between 25 °C and 27 °C. In the highlands and the Saharan Atlas, the temperatures recorded a thermal amplitude higher than that of the coastal regions, given by an average daily temperature in January which is around 2 °C and 9 °C. However, in July these are between 19 °C and 33 °C. 

Spatial variation maps displaying the maximum and the minimum altitudes observed in 16 watersheds in northern Algeria are shown in Figure 1B,C, respectively. The maps show that the northern Algeria morphology is higher on the southern side than on the northern, where the minimum altitudes observed on the marine side reach measurements between 17.9 m and 271 m in the basins numbered 3, 2, and 4. However, the maximum altitudes in this region are between 262.2 m and 1079 m. In the southeastern region, the maximum elevations of basins numbered 06, 05, 07, and 12 almost reach values between 1080 m and 1896 m. Contrariwise, in the middle region of basins numbered 6 and 7, the highest points in this region arrive between 1897 m and 2304 m. However, the minimum altitudes are between 524 m and 1280 m. In the western area, the maximum altitudes reach the interval values given between 1080 m and 1487 m, especially in basins 08, 11, 16, and 01. Where the minimum elevations are between 272 m and 523 m.

The hydrometric network of northern Algeria consists of 200 measuring stations, which are distributed over 16 watersheds. The present work aims to estimate the runoff of 102 sub-basins obtained by defining the extreme outlets of the wadis in the entire region. In the middle location of each sub-basin, we chose a meteorological and a hydrological station in the same coordinate to provide sufficient data that were used to assess rainfall flow between 1965 and 2020. The climatic data used in this study are precipitation (IAR) and temperature (IAT). The data was obtained from the available dataset on the climatic stations provided by the National Centers for Environmental Information (NCEI-NOAA). https://doiwww.ncdc.noaa.gov/ (accessed on 12 May 2021), and Climate Knowledge Portal https://climateknowledgeportal.worldbank.org/ (accessed on 17 May 2021), whereas the hydrological and geographical data, which are the watershed area (S) and the length of the watercourse (WC), were obtained from hydrometric stations provided by the Algerian National Agency of Hydrological Resources (A.N.R.H).

The De Martonne aridity index (I) was used in this study as input data of the dynamic model given by the artificial neuron network (ANN_2_) and to classify the bioclimatic floor of the northern Algeria area. The I index values were obtained according to the following Equation (1) [29]:

(1) $$I=\frac{\mathrm{IAR}}{\mathrm{IAT}+10}\;$$

The actual measurement of inter-annual rainfall-runoff (IARR_R_) was obtained from the A.N.R.H service. This data was used to estimate the computational residues (IARR’) obtained by Ol’Dekop’s model and to control the performance of the proposed model in the study area [21], that is:

(2) $${\;\mathrm{IARR}}_{E}=\mathrm{IAR}-{\mathrm{IAE}}_{a}\;$$

(3) $${\mathrm{IARR}}^{\prime }={\mathrm{IARR}}_{R}-{\mathrm{IARR}}_{E}\;$$

where IARR_E_ represents the inter-annual rainfall-runoff data estimated by the Ol’Dekop model, IAEa is the mean annual real evapotranspiration, and IARR_R_ is the inter-annual real rainfall-runoff.

### 2.2. Artificial Neuron Network

An artificial neuron network (ANN) is a data-driven process with a flexible mathematical algorithm capable of solving the complex nonlinear relationships between input and output datasets. In fact, it mimics the biological neuron architecture [30]. It is a family of parallel architectures used to solve the most complex mathematical problems in modeling, optimization, and prediction [31,32]. In practice, using ANN involves taking into consideration three main elements:

- The interconnection between the input data ensures good results through the process.
- The transfer function controls the generation of the neural output.
- The summing function and the statistical parameters describe how the weights of input data are adjusted during the treatment [30].

During computation the ANN receives data from the input layer, then a combination between selected data is performed by the hidden layer using the summing function and a number of statistical control parameters. In general, the summing function formula is represented by:

(4) $${\mathrm{net}}_{j}=\sum^{\;}W_{\mathrm{ij}}x_{i}$$

where net_j_ is the mean of weighted input for the jth neuron; W_ij_ is the weight from the ith neuron in the previous layer to the jth neuron in the current layer, and X_i_ is the input from the ith to the jth neuron.

The transfer function Ψ is also used by the hidden layer to generate the final result, which is called by output data Y_j_, using the function (5). The training stops when the error obtained by the validation test reaches the minimum.

(5) $$Y_{j}=\Psi \left({\mathrm{net}}_{j}+\theta_{j}\right)$$

where net_j_ represents the obtained data from the summing function (4), θ_j_ is the external threshold that is obtained from the summing step. In literature, hydro-climatic modeling using ANN typically uses the feed-forward network structure with either one or multiple layers, depending on the objective of the study [33,34]. When using ANN for modeling climatic and hydrological phenomena, a set of fundamental decisions need to be made [33], which are:

- The choice to use the appropriate neural network architecture.
- The best selection of a suitable training algorithm, study periods, and network structure.
- The way to pre-process and post-process the input and output data, respectively.

#### ANN Transfer Function

In the experimental part, the transfer function used by the ANN model to estimate the output data was given by the trend equation of the multiple linear regression model (MLR). This latter one was applied to a set of input data (X_i_*) that could prove a good regression with IARR’*. The test was conducted after linearizing the data series using the Ln equation. In this machine learning, the output data represents the predicted residual values obtained by the Ol’Dekop model. The concept of multiple linear regression used to study the linear relationship between the dependent variable Y and the vector of regressors (X, X_2_, …, X_k_) is given by the following function [35]:

(6) $$Y=\alpha +\beta_{1}X_{1}+\beta_{2}X_{2}+\ldots +\beta_{k}X_{k}+Ɛ$$

where α is the intercept, β is the slope or the coefficient, k is the number of observations and Ɛ represents the estimation error.

### 2.3. Water Balance Model

A set of nonparametric and parametric models used to compare and analyze the performance of the proposed model are detailed by the Equations (7)–(11), as detailed next.

#### 2.3.1. Schreiber

Schreiber [7] proposed a simple model to estimate inter-annual evapotranspiration (IAEa) in terms of inter-annual precipitation (IAR) and mean annual potential evapotranspiration (IAEo), that is:

(7) $$\mathrm{IAEa}=\mathrm{IAR}\times \left[1-\exp\left(-\frac{{\mathrm{IAE}}_{o}}{\mathrm{IAR}}\right)\right]$$

#### 2.3.2. Ol’Dekop

Ol’Dekop [36] applied a trigonometric hyperbolic tangent function (8) to show the relationship between potential evapotranspiration (IAE_o_) and the drying factor ($\frac{\mathrm{IAR}}{{\mathrm{IAE}}_{0}}$).

(8) $${\mathrm{IAE}}_{a}={\mathrm{IAE}}_{0}\times \tanh\left(\frac{\mathrm{IAR}}{{\mathrm{IAE}}_{0}}\right)\;$$

#### 2.3.3. Budyko

Budyko [9] defined an average estimation using Schreiber [7] and Ol’dekop [8] models to reduce errors obtained by both models (9), which is given by following formula,

(9) $${\mathrm{IAE}}_{a}={\left[\mathrm{IAR}\times \left[1-\exp\left(-\frac{{\mathrm{IAE}}_{o}}{\mathrm{IAR}}\right)\right]\times \mathrm{ETR}-\tan h\left(\frac{\mathrm{IAR}}{{\mathrm{IAE}}_{o}}\right)\right]}^{0.5}$$

#### 2.3.4. Yang

Yang [37] proposed an alternative model (10) to assess mean annual actual evapotranspiration by entering local parameter (n) characteristics on Budyko’s hypothesis. The model uses the watershed characteristics for a better estimation, that is

(10) $${\mathrm{IAE}}_{a}=\left[{[\left[{\left(\frac{{\mathrm{IAE}}_{o}}{\mathrm{IAR}}\right)}^{-n}\right]+1]}^{-\frac{1}{n}}\right]\times I$$

where n > 0.

#### 2.3.5. Sharif

This model is an improvement of the Mezentsev–Choudhury–Yang (MCY) proposition, achieved by replacing b, k, and n parameters with 0, 2, and 1, respectively [38].

(11) $${\mathrm{IAE}}_{a}=\frac{2\times \mathrm{IAR}\times {\mathrm{IAE}}_{o}}{\mathrm{IAR}+2\times {\mathrm{IAE}}_{o}}$$

where by IAR is the inter-annual rainfall; IAE_0_ is inter-annual potential evapotranspiration; and IAE_a_ is inter-annual actual evapotranspiration.

### 2.4. Metrics of Performance

The statistical criteria applied in each modeling step, which are also used to compare the performance of the obtained model with non-parametric and parametric models mostly cited in the literature, are well detailed in Table 1. Where by Qs is the estimated runoff; Qo is the observed runoff; N is the total number of ordinates; K is the number of independent variables; and e_i_ is the residual for the time period i.

## 3. Results

In this section, we start our study by spatially analyzing and classifying the input data used by each sub-model. Then we show the ANN design, such as the choice of input data by the selection criteria, the regression equation used as a transfer function, and the output results, which are mainly used to estimate the residual data (IARR’). Finally, the computational and calibration steps followed to obtain the runoff equation are given in detail in the last part.

### 3.1. Data Description and Classification

**Figure 2:**
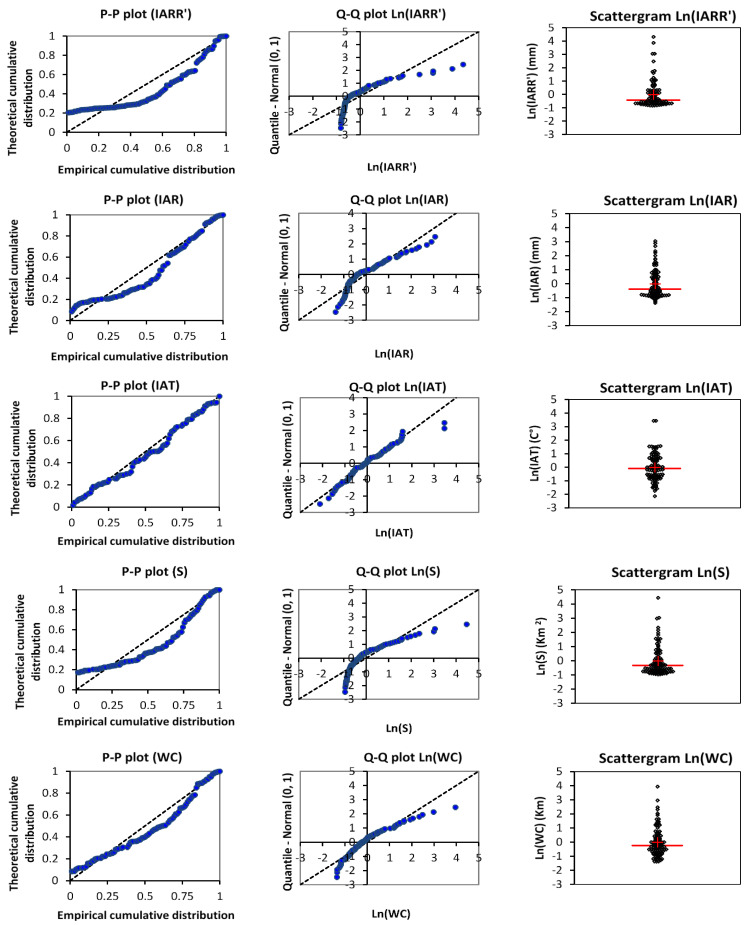
P–P plot, Q–Q plot, and scattergram graph of the dataset series, used in this modeling. Inter-annual rainfall-runoff residual (IARR’), inter-annual rainfall (IAR), inter-annual temperature (IAT), watershed area (S), and watercourse (WC).

Data distribution and variability analyses of selected variables used for modeling IARR’ are shown in this section, which aims to study the behavior and the regression relationship between input variables and the IARR’ dataset. This study is based mainly on qualitative and quantitative tests, where the results of the data distribution are given by P–P plot, Q–Q plot (which allowed us to compare the empirical and theoretical distribution of data and cumulative quartiles, respectively, using normal law), and scattergram graph (Figure 2). The Q–Q plot and the scattergram graph are obtained after linearizing the used variables by applying the Ln function, to simplify the comparison between data series distributions, which have different measurement units. Moreover, Table 2 gives us a set of statistical parameters to quantify the variability of the data series. The P–P plot and the Q–Q plot show that IAR, S, and WC data series have a similar variability to the IARR’s dataset. However, IAR and S data distributions are closer to IARR than the WC data series (Figure 2). On the other hand, IAT shows a different behavior of data distribution, where the graphs prove that the stochastic model, which defines the IAT variability, is closer to the normal law. The *p*-value results obtained by the D_KS_ fit test using a set of distribution laws (Table 2) show that IARR’ and IAR follow the Weibull 3 law, where the *p*-values equal 0.9789 and 0.8572, respectively. On contrary, IAT data have a GEV distribution, where the *p*-value is equal to 0.8764. Moreover, S and WC show a similar distribution, in which both variables follow the law of gamma 2. According to the fit results, the last three variables accept the Weibull 3 as a second closest fit model, where *p*-values equal 0.6136, 0.8363, and 0.8263, respectively. In Figure 2, the scattergram shows a descriptive comparison between the variability of data cited above. The graphs show a similar variability between IARR’, IAR, and S data series, where the majority of the values of each variable are very close between them and below the average of their series (which equals 49.113, 494.6529, and 719.7106, respectively). On the other hand, Table 2 shows that 25% of the dataset, which is bounded between the third quartile and the maximum value, has very large variability, given by the interval of [64.3229, 284.3221], [607.00, 1107.00], and [1028.25, 4050.00], respectively. The WC variable shows a slight difference between data distribution. Inversely, the variability is more similar to IARR’. In this series, the mean and the median are close, and equal 45.60 and 51.10, respectively. However, 25% of the WC values give a very high variability, which is given by a value range of [65.5250, 179.80]. On the other hand, the IAT dataset shows a very low variability given by a variation coefficient of 0.1180. This last dataset proves a convergence between the median and the mean, which are equal to 15.2792 and 15.4569, respectively. Moreover, the scattergram shows that the upper and the lower IAT values, compared to the average, have a similar distance, given by the interval of [−2, 0] and [0, 2], respectively. In Table 2, this similarity is given by [11.5833, 15.2792] and [15.2792, 21.7583], respectively. According to this analysis, the greatest variability was obtained from IARR’ and S datasets, whereby the variation coefficients equal 1.1039 and 1.0379, respectively.

**Figure 3:**
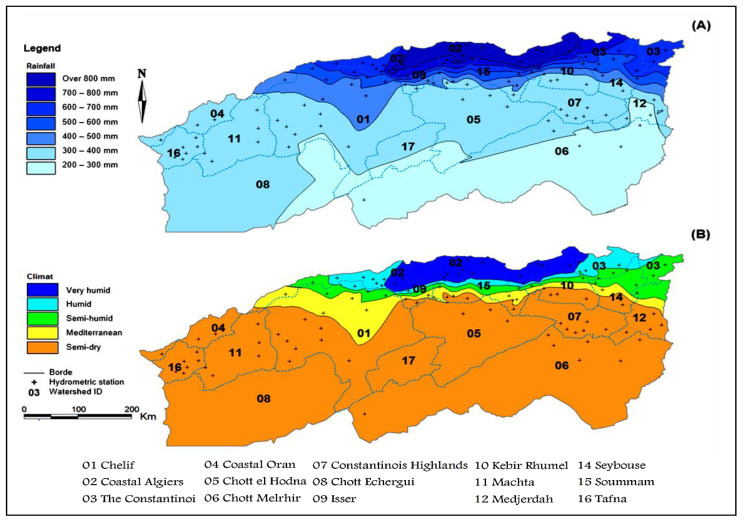
Map of spatial inter-annual rainfall distribution (**A**), followed by bioclimatic classification (**B**) of northern Algeria.

The spatial inter-annual rainfall distribution and the De Martonne index obtained by applying Equation (1) are mapped using the data of 102 meteorological stations to determine the bioclimatic floor of each watershed of northern Algeria between 1965 and 2020. This study helps to provide the application areas used to control the performance of the model proposed in this section. Climate classification analysis is shown in Figure 3. A very large variability of rainfall from north to south is shown in Map (a), wherein the southern part, the IAR reaches up to 200 mm. However, in the north, the rainfall reaches values higher than 800 mm.

According to the bioclimatic classification given by [45], northern Algeria has a climatic diversity spread over five floors, from very humid to very dry (Figure 3B). The figure shows that watershed 02, which is located in the northern part and overlooking the Mediterranean Sea has a very humid climate, characterized by IAR values varying between 700 mm and >800 mm (Figure 3A). On the other hand, watersheds 09, 15, and 10 have the greatest climatic diversity, varying between very humid, humid, semi-humid, Mediterranean, and semi-dry. Where in this area, the rainfall varies between 400 mm and >800 mm. This diversity depends mainly on the geographical and geological characteristics of the region. In the northeastern part, watershed 03 is characterized by a very humid, humid, and semi-humid climate, where the IAR varies between 500 mm and >800 mm. The catchment area 01, 14, and 12 have a climatic diversity which is between humid, semi-humid, Mediterranean, and semi-dry. In this area, the minimum rainfall was observed in watershed 14, which arrives at 300 mm. Differently, in watersheds 01 and 12, the minimum rainfall values reach up to 200 mm in the southern areas.

In addition, watersheds 16, 04, 11, 08, 17, 05, 06, and 07 are characterized by a semi-dry climate, where the rainfall varies generally between 300 mm and 400 mm. Contrariwise, the minimum rainfall of watersheds 08, 17, 05 and 06 reaches up to 200 mm.

### 3.2. Proposed ANNs

**Figure 4:**
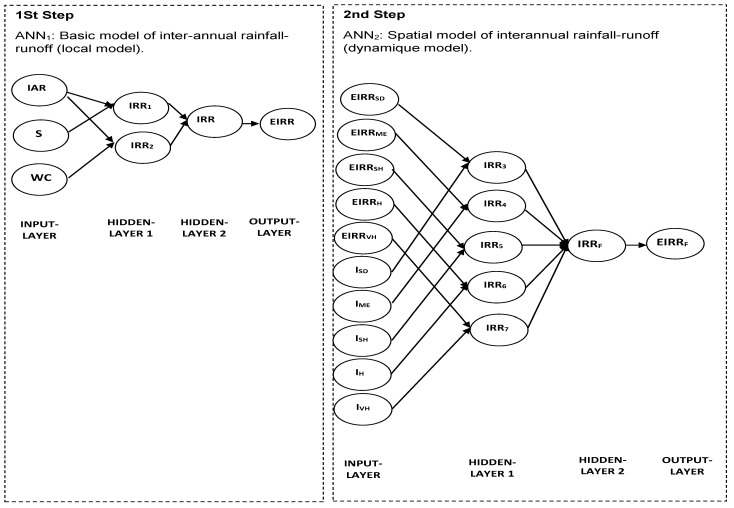
Proposed ANN_s_ model architecture, used to estimate residuals computational of the Ol’Dekop model. The local and basic model (ANN_1_), general and dynamic model (ANN_2_). Inter-annual rainfall (IAR), watershed area (S), watercourse (WC), De Martonne index (I), Semi-Dry (SD), Mediterranean (ME), Semi-Humid (SH), Humid (H), and Very Humid (VH).

The different steps followed to obtain the best ANNs model for estimating the computational errors of IARR’ given by the Ol’Dekop model are represented in this section. This study allows us to develop a new form of water balance model based on a set of climatic and geomorphological variables, which can be applied in a different area, without being conditioned by the aridity state of the watershed. The statistical tests used in this study are: residual analysis curve, R^2^, R^2^_Adj_, MSE, RMSE, and Durbin–Watson (DW). These latter tests were used to analyze the performance of each transfer equation used by the ANN model, and also to quantify the reliability degree of the proposed model compared to a set of parametrical and non-parametrical water balance models, which are what is mostly used in the literature. Our model is classified into two steps, given as ANN_1_ and ANN_2_ (Figure 4), which show that initially a local model (ANN_1_) was given to estimate IARR in all northern Algeria watersheds. Then an improvement was made to increase the reliability of the previous model in each basin climatic area and to make it more dynamic and applicable in different regions (ANN_2_).

Figure 4 shows that the two previous sub-models are the type of feed-forward network with the architecture of (3-2-1-1) and (10-5-1-1), respectively. In the first attempts of this modeling, we used IAR, S, and WC variables as input layers in the ANN_1_ model. After that, we classified the estimation results that were obtained by this model in groups, according to each bioclimatic level. In this step, we also used the aridity index *I* of each hydrological station as input data in the ANN_2_ model to determine, in each climatic area, its transfer function, which allows us to deduce the final rainfall-runoff model. Figure 4, shows that the intermediate nodes (denoted by IRR_1_, IRR_2_, IRR_3_, IRR_4_, IRR_5_, IRR_6_, and IRR_7,_ respectively) make it possible to apply sub-processing, using a summing and transfer function on the input data “output layer” to estimate the output data in each step of the ANN model. In our case, the summing function combines the input variables two by two to have the best modeling results. Moreover, the choice of combination between variables was justified by the results of the correlation test, which were applied to the linearized variables compared to the linearized IARR’ (IARR’*) (Table 3). The table shows the degree of correlation between a set of candidate variables that were cited in the previous section of this study and IARR’*, using two correlation forms. A direct correlation was found between the IARR’* and (S*, WC*, IAT*, IAR*, and I*), then an indirect correlation between IARR’* and (WC*, S*), by studying the relationship between IARR’* and (($\frac{\mathrm{IARR}\prime *}{\;\mathrm{WC}*}$), ($\frac{\mathrm{IARR}\prime *}{\;S*}$)) then between (($\frac{\mathrm{IARR}\prime *}{\;\mathrm{WC}*}$), ($\frac{\mathrm{IARR}\prime *}{\;S*}$)) and (Wc*, S*), respectively.

Table 3 shows that IARR’* has the best correlation with IAR*, which equals 0.8513. It is also strongly correlated with climatic data obtained from the I* index. Contrariwise, the IAT* shows a weak correlation. However, the geo-hydrologic variables such as WC* and S* are slightly correlated with the response variable (IARR’*), which equals 0.5141 and 0.5023, respectively. The results highlight that the relation proposed by ($\frac{\mathrm{IARR}\prime *}{\;\mathrm{WC}*}$) and ($\frac{\mathrm{IARR}\prime *}{\;S*}$) shows a very good correlation with IARR’*, given by 0.8124 and 0.7322, respectively. Furthermore, the new variables also show a strong correlation with WC* and S*, respectively, given by correlation coefficients of 0.6168 and 0.7923, respectively.

### 3.3. IARR Modeling

**Figure 5:**
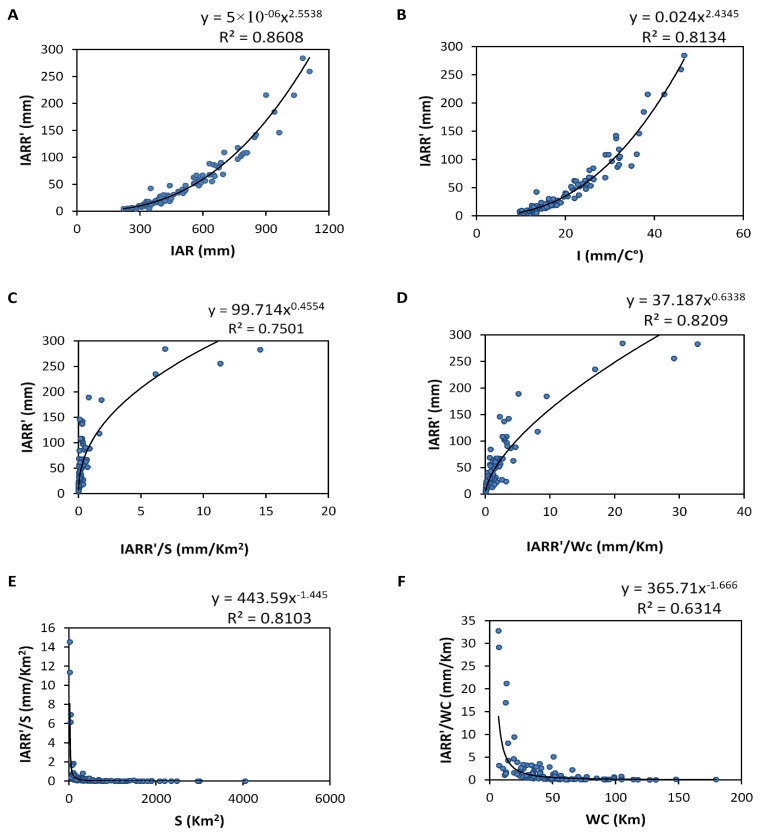
Nonlinear regression curve between inter-annual rainfall-runoff residuals data obtained by Ol’Dekop’s model (IARR’) and ANN_S_ input variables which are (**A**) Inter-annual rainfall (IAR); (**B**) De martone index (I); (**C**,**E**) watershed area (S); (**D**,**F**) watercourse (WC).

Describing different modeling steps used to propose a new equation of IARR estimation is based mainly on the computational error analysis of the Ol’Dekop model in a set of bioclimatic floors. A non-linear regression relationship between IARR’ and selected input variables provided in (Table 3) are shown graphically in Figure 5 as the first step of this analysis. In this regard, a direct regression is applied between the response variable (IARR’) and the input variables (IAR, I). Then, intermediate variables were used to express the indirect relationship between (S, Wc) and IARR’. The results show a similar regression for each pair of the dataset (IARR ‘, IAR) and (IARR’, I), given by an R^2^ which equals 0.8608 and 0.8134, respectively.

The graphs shown in Figure 5A,B prove some trends of values, which are shown in the range of the maximum value. Regression models cited above are defined by Equations (12) and (13):

(12) $$\mathrm{IARR}\prime =5\times 10^{-6}\times {\mathrm{IAR}}^{2.5538}$$

(13) $$\mathrm{IARR}\prime =0.024\times I^{2.4345}$$

On the other hand, the figure also shows a good nonlinear regression between IARR’ and$\;\frac{\mathrm{IARR}\prime }{S}$, and also between IARR’ and $\frac{\mathrm{IARR}\prime }{\mathrm{Wc}}$ according to R^2^ results, which equal 0.7501 and 0.8209, respectively, given by Figure 5C,D. The regression relationship between both input variables are defined by Equations (14) and (15), respectively:

(14) $$\mathrm{IARR}\prime =99.714\times {\left(\frac{\mathrm{IARR}\prime }{S}\right)}^{0.4554}$$

(15) $${\mathrm{IARR}}^{\prime }=37.187\times {\left(\frac{\mathrm{IARR}\prime }{\mathrm{Wc}}\right)}^{0.6338\;}\;$$

Moreover, both ratios ($\frac{\mathrm{IARR}\prime }{S}$) and ($\frac{\mathrm{IARR}\prime }{\mathrm{Wc}}$) proved a good regression trend with S and WC variables, respectively. Where R^2^ equals 0.8103 and 0.6314, respectively. Both statistical relationships between input and response variables are defined by Equations (16) and (17):

(16) $$\frac{\mathrm{IARR}\prime }{S}=443.59\times S^{-1.445}$$

(17) $$\frac{\mathrm{IARR}\prime }{\mathrm{Wc}}=365.71\times {\mathrm{Wc}}^{-1.666}$$

In this study, we found that all the cases of regression cited in Figure 5 followed the power model trend.

**Figure 6:**
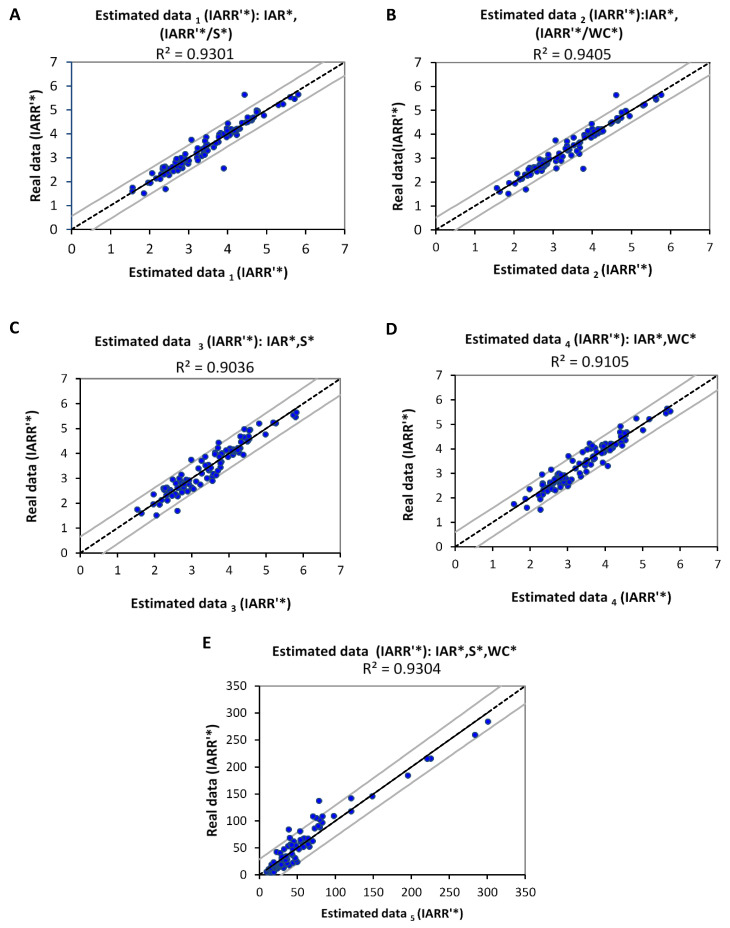
Multiple linear regression curves between real and estimated Ol’Dekop’s rainfall-runoff residual data (IARR’), obtained by ANN_1_ transfer functions by using a set of input variables which are (**A**) (IAR*, IARR*/S*); (**B**) (IAR*, IARR*/WC*); (**C**) (IAR*, S*); (**D**) (IAR*, WC*); (**E**) (IAR*, S*, WC*). * input variable linearized by the Ln function, as follow: X_i_* = Ln X_i_. Inter-annual rainfall (IAR), watershed area (S), watercourse (WC).

Figure 6 represents the linear regression graphs, which express the degree of correlation between the real values of IARR’* and the estimated values that were obtained by the IRR_1_ and IRR_2_ models. In this step, we proved the correlation’s degree and the reliability of the obtained model. We have well explained the different multiple regression models, which are applied to the pair of variables (IAR*, S*) and (IAR*, WC*), using the intermediate variable ($\frac{\mathrm{IARR}\prime *}{\;S*}$) and ($\frac{\mathrm{IARR}\prime *}{\;\mathrm{WC}*}$), respectively, which showed a good nonlinear regression with S and WC data, and also with IARR’ (Figure 5). Moreover, Table 4 shows a set of statistical parameters relating to this modeling, which gives information about the reliability and the trend analysis of the sub-models that are noted by (A, B, C, and D), compared to the linearized real data (IARR’*). According to the results, we found that the regression model obtained from (IAR*, $\frac{\mathrm{IARR}\prime *}{\;S*}$) and (IAR*, $\frac{\mathrm{IARR}\prime *}{\;\mathrm{WC}*}$) have a very good regression, proved by an (R^2^, R^2^_adj_) results which equal (0.9301, 0.9286) and (0.9405, 0.9393), respectively. Moreover, the errors given by MSE, RMSE, and DW show that all models did not prove a large trend deviation compared to IARR’* real data. The computational steps of IRR_1_ and IRR_2_ models are well detailed by the following Equations (18) and (19):

(18) $$\mathrm{Ln}\left({\mathrm{IRR}}_{1}\right)=1.7166\times \mathrm{Ln}\left(\mathrm{IAR}\right)+0.2057\times \mathrm{Ln}\left(\frac{\mathrm{IARR}\prime }{S}\right)-6.5738$$

(19) $$\mathrm{Ln}\left({\mathrm{IRR}}_{2}\right)=1.4538\times \mathrm{Ln}\left(\mathrm{IAR}\right)+0.3296\times \mathrm{Ln}\left(\frac{\mathrm{IARR}\prime }{\mathrm{Wc}}\right)-5.3946\;$$

To deduce the previous equations as functions of fundamental variables IAR, S, and WC, we start with Equation (18) by replacing ($\frac{\mathrm{IARR}\prime }{S}$) with (S) using Equation (16). So we obtain:

(20) $$\mathrm{Ln}\left({\mathrm{IRR}}_{1}\right)=1.7166\times \mathrm{Ln}\left(\mathrm{IAR}\right)+0.2057\times \mathrm{Ln}\left(443.59\times S^{-1.445}\right)-6.5738\;\;=1.7166\times \mathrm{Ln}\left(\mathrm{IAR}\right)-0.2972\times \mathrm{Ln}\left(S\right)-5.3201$$

On the other hand, we use Equation (17) to replace ($\frac{\mathrm{IARR}\prime }{\mathrm{Wc}}$) with (WC) in Equation (19). We find:

(21) $$\mathrm{Ln}\left({\mathrm{IRR}}_{2}\right)=1.4538\times \mathrm{Ln}\left(\mathrm{IAR}\right)+0.3296\times \mathrm{Ln}(365.71\times {\mathrm{Wc}}^{-1.666})\;\;\;\;\;=1.4538\times \mathrm{Ln}\left(\mathrm{IAR}\right)-0.5491\times \mathrm{Ln}\left(\mathrm{Wc}\right)-3.4494$$

Figure 6C,D shows the reliability of the results obtained by the IRR_1_ and IRR_2_ models that are given by Equations (18) and (19), respectively. The corresponding graphs show a good fit of regression between the actual and the estimated values of IARR’*. This reliability was shown by R^2^, and R^2^_Adj_ statistical parameters, which equal (0.9036, 0.9006) and (0.9105, 0.909), respectively (Table 4). We apply the Exp function in Equations (20) and (21), to obtain the IRR model used by ANN_1_.

We have,

(22) $${\mathrm{IRR}}_{1}=\mathrm{Exp}\left(1.7166\times \mathrm{Ln}\left(\mathrm{IAR}\right)-0.2972\times \mathrm{Ln}\left(S\right)-5.3201\right)\;\;=0.0049\times \left({\mathrm{IAR}}^{1.7166}\right)\times \left(S^{-0.2972}\right)$$

(23) $${\;\mathrm{IRR}}_{2}=\mathrm{Exp}\left(1.4538\times \mathrm{Ln}\left(\mathrm{IAR}\right)-0.5491\times \mathrm{Ln}\left(\mathrm{Wc}\right)-3.4494\right)\;\;=0.0318\times \left({\mathrm{IAR}}^{1.4538}\right)\times \left({\mathrm{Wc}}^{-0.5491}\right)$$

In this step, we found two reliable equations to estimate IARR’. Figure 6E shows that the best regression can be obtained as a function of the three variables (IAR, S, and WC), which is given by Equation (24).

According to Equations (22) and (23), the general model IRR is defined as follows:

(24) $$\mathrm{IRR}=\sqrt{{\mathrm{IRR}}_{1}\times {\mathrm{IRR}}_{2}}\;\;\;\;\;\;\;\;\;\;\;\;\;\;\;\;\;\;\;\;\;\;\;\;\;\;\;\;\;\;\;\;\;\;\;\;\;\;\;\;\;\;\;\;\;\;\;\;\;\;\;\;\;\;\;\;\;\;\;\;\;\;\;\;\;\;\;\;\;\;\;\;\;\;\;\;\;\;\;\;\;\;\;\;\;\;\;\;\;\;\;\;\;\;\;\;\;\;\;\;\;\;\;\;\;\;\;\;=\sqrt{0.0049\times \left({\mathrm{IAR}}^{1.7166}\right)\times \left(S^{-0.2972}\right)\times 0.0318\times \left({\mathrm{IAR}}^{1.4538}\right)\times \left({\mathrm{Wc}}^{-0.5491}\right)}\;\;\;\;\;\;\;\;\;\;\;\;\;\;\;\;\;\;\;\;\;=0.01\times \left({\mathrm{IAR}}^{1.5852}\right)\times \left(S^{-0.1486}\right)\times \left({\mathrm{Wc}}^{-0.27455}\right)$$

Table 4 shows that the IRR model gives a good estimation, proven by a set of statistical parameters. The results obtained by this model give an R^2^ and R^2^_Adj_, which are equal to 0.9518 and 0.9508, respectively. Moreover, the computational errors given by MSE, RMSE, and DW parameters show values of 208.539, 11.4409, and 0.7094, respectively. The ANN_1_ model shows that the use of geomorphological parameters increases the reliability of estimation when compared with the simple regression model obtained by IAR only (Figure 5A).

In the second step of this study, we improved the proposed model, which is defined by Equation (24) to be more dynamic and applicable to each bioclimatic region. We applied multiple linear regression to aridity index series (I) and the predicted data obtained previously by the IRR model. This technique was applied separately to each bioclimatic stage in order to find the corresponding estimation model of each area. Table 5 shows all the statistical parameters relating to each local regression model. The results show that the model obtained in a very humid climate area gives a more reliable estimation compared to the performance models of other bioclimatic floors, where the R^2^ and R^2^_Adj_ given for the IRR_7_ model equal 0.9072 and 0.9001, respectively (Figure 4 and Table 5). The table shows that the obtained models have different reliability in each climate region, wherein the Mediterranean area, the R^2,^ and R^2^_Adj_ were proven to perform well, and equal 0.7804 and 0.7647, respectively.

On the other hand, in the semi-dry, semi-humid, and humid climate floor, the R^2^, R^2^_Adj_ equal (0.6501, 0.6420), (0.6820, 0.6675) and (0.6533, 0.6448), respectively. Moreover, the trend pattern obtained by modeling the IARR’ in the dry climate floors is increased when compared to the estimated IARR’ in wet regions. The performance criteria show that the greatest values are given in the semi-dry climate level, where MSE, RMSE, and DW equal 0.0783, 0.298, and 1.6870, respectively. In the humid area, R^2^ proved lower performance compared to the estimation obtained in the Mediterranean region. Contrariwise, the errors are more remarkable in the humid regression model, where MSE, RMSE, and DW values equal 0.0138, 0.1175, and 0.8625, respectively. On the other hand, on the Mediterranean climate floor, these parameters equal 0.0296, 0.1721, and 1.7861, respectively. The model named IRR_3_, IRR_4_, IRR_5_, IRR_6_, and IRR_7_, which were obtained by modeling IARR’ in semi-dry, Mediterranean, semi-humid, humid, and very humid climate floors, respectively, are defined by Equations (25)–(29), as follows:

(25) $$\mathrm{Ln}\left({\mathrm{IRR}}_{3}\right)=0.50033\times \mathrm{Ln}\left({\mathrm{EIRR}}_{S}\right)+1.16331\times \mathrm{Ln}\left(I_{S}\right)-1.74497$$

(26) $$\mathrm{Ln}\left({\mathrm{IRR}}_{4}\right)=0.42496\times \mathrm{Ln}\left({\mathrm{EIRR}}_{\mathrm{ME}}\right)+1.17641\times \mathrm{Ln}\left(I_{\mathrm{ME}}\right)-1.41471$$

(27) $$\mathrm{Ln}\left({\mathrm{IRR}}_{5}\right)=0.29175\times \mathrm{Ln}\left({\mathrm{EIRR}}_{\mathrm{SH}}\right)+1.00656\times \mathrm{Ln}\left(I_{\mathrm{SH}}\right)-0.2404$$

(28) $$\mathrm{Ln}\left({\mathrm{IRR}}_{6}\right)=-0.16179\times \mathrm{Ln}\left({\mathrm{EIRR}}_{H}\right)+1.34092\times \mathrm{Ln}\left(I_{H}\right)+0.45666$$

(29) $$\mathrm{Ln}\left({\mathrm{IRR}}_{7}\right)=0.28185\times \mathrm{Ln}\left({\mathrm{EIRR}}_{\mathrm{VH}}\right)+1.50295\times \mathrm{Ln}\left(I_{\mathrm{VH}}\right)-1.60261$$

We apply the Exp function on Equations (25)–(29)to generate the final estimation model (IRR_F_) in terms of IAR, S, WC, and I (Table 5) So we find,

(30) $$\;\;\;\;{\mathrm{IRR}}_{3}=0.1747\times {\mathrm{EIRR}}_{S}{}^{0.50033}\times I_{S}{}^{1.16331}$$

(31) $${\mathrm{IRR}}_{4}=0.2430\times {\mathrm{EIRR}}_{\mathrm{ME}}{}^{0.42496}\times I_{\mathrm{ME}}{}^{1.17641}$$

(32) $${\mathrm{IRR}}_{5}=0.7865\times {\mathrm{EIRR}}_{\mathrm{SH}}{}^{0.29175}\times I_{\mathrm{SH}}{}^{1.00656}\;$$

(33) $$\;\;\;\;\;\;{\mathrm{IRR}}_{6}=1.5788\times {\mathrm{EIRR}}_{H}{}^{-0.16179}\times I_{H}{}^{1.34092}$$

(34) $$\;\;\;\;\;\;\;{\mathrm{IRR}}_{7}=0.2014\times {\mathrm{EIRR}}_{\mathrm{VH}}{}^{0.28185}\times I_{\mathrm{VH}}{}^{1.50295}$$

We apply the different models obtained in each bioclimatic area using all the datasets of northern Algeria to show the trend that can be caused by the static models. In this step, we want to propose a dynamic model, by eliminating all constants and finding a mathematical relationship with variables that can prove a good correlation. Table 6 shows statistic results, obtained from IRR_3_, IRR_4_, IRR_5_, IRR_6_, IRR_7_, and IRR_F_ models. The table shows that all previous models cited above proved an R^2^ and R^2^_Adj_ greater than 0.80. Moreover, the error trends increase in these models, which are proven by MSE, RMSE, and DW parameters. On the other hand, the IRR_F_ model has the best reliability, given by an R2, which equals 0.9841. This model proved a low tendency, where the MSE, RMSE, and DW equal 62.5948, 5.9117, and 0.5250, respectively. The final model (IRR_F_) was obtained by applying the weighted average using the R^2^ values that are obtained in Table 6 as weighting coefficients to estimate the a1 and a2 coefficients of this model. Where the IRR_F_ equation is defined as follows:

(35) $${\mathrm{IRR}}_{F}={\mathrm{Cte}\times \mathrm{EIRR}}^{a1}{\times I}^{a2}$$

We have,

(36) $$\;a1=\frac{\left(R_{{\mathrm{IRR}}_{3}}^{2}\times a1_{{\mathrm{IRR}}_{3}}\right)+\left(R_{{\mathrm{IRR}}_{4}}^{2}\times a1_{{\mathrm{IRR}}_{4}}\right)+\left(R_{{\mathrm{IRR}}_{5}}^{2}\times a1_{{\mathrm{IRR}}_{5}}\right)+\left(R_{{\mathrm{IRR}}_{6}}^{2}\times a1_{{\mathrm{IRR}}_{6}}\right)+\left(R_{{\mathrm{IRR}}_{7}}^{2}\times a1_{{\mathrm{IRR}}_{7}}\right)}{R_{{\mathrm{IRR}}_{3}}^{2}+R_{{\mathrm{IRR}}_{4}}^{2}+R_{{\mathrm{IRR}}_{5}}^{2}+R_{{\mathrm{IRR}}_{6}}^{2}+R_{{\mathrm{IRR}}_{7}}^{2}}$$

By replacing variables with values, we find:

$$a1=\frac{\begin{array}{c} \left(0.9494\times 0.50033\right)+\left(0.9538\times 0.42496\right)+\left(0.9443\times 0.29175\right)+\left(0.8358\times -0.16179\right)\\ +\left(0.9616\times 0.28185\right) \end{array}}{\left(0.9494+0.9538+0.9443+0.8358+0.9616\right)}\;=0.27808\;\;\;\;\;\;\;\;\;\;\;\;\;\;\;\;\;\;\;\;\;\;\;\;\;\;\;\;\;\;\;\;\;\;\;\;\;\;\;\;\;\;\;\;\;\;\;\;\;\;\;\;\;\;\;\;\;\;\;\;\;\;\;\;\;\;\;\;\;\;\;\;\;\;\;\;\;\;\;\;\;\;\;\;\;\;\;\;\;\;\;\;\;\;\;\;\;\;\;\;\;\;\;\;\;\;\;\;\;\;\;\;\;\;\;\;\;\;\;\;\;\;\;\;\;\;\;\;\;\;\;$$

We have also,

(37) $$\;a2=\frac{\left(R_{{\mathrm{IRR}}_{3}}^{2}\times a2_{{\mathrm{IRR}}_{3}}\right)+\left(R_{{\mathrm{IRR}}_{4}}^{2}\times a2_{{\mathrm{IRR}}_{4}}\right)+\left(R_{{\mathrm{IRR}}_{5}}^{2}\times a2_{{\mathrm{IRR}}_{5}}\right)+\left(R_{{\mathrm{IRR}}_{6}}^{2}\times a2_{{\mathrm{IRR}}_{6}}\right)+\left(R_{{\mathrm{IRR}}_{7}}^{2}\times a2_{{\mathrm{IRR}}_{7}}\right)}{R_{{\mathrm{IRR}}_{3}}^{2}+R_{{\mathrm{IRR}}_{4}}^{2}+R_{{\mathrm{IRR}}_{5}}^{2}+R_{{\mathrm{IRR}}_{6}}^{2}+R_{{\mathrm{IRR}}_{7}}^{2}}$$

We replace variables with values, we find:

$$a2=\frac{\begin{array}{c} \left(0.9494\times 1.16331\right)+\left(0.9538\times 1.17641\right)+\left(0.9443\times 1.00656\right)+\left(0.8358\times 1.34092\right)+\\ \left(0.9616\times 1.50295\right) \end{array}}{\left(0.9494+0.9538+0.9443+0.8358+0.9616\right)}\;=1.2364\;\;\;\;\;\;\;\;\;\;\;\;\;\;\;\;\;\;\;\;\;\;\;\;\;\;\;\;\;\;\;\;\;\;\;\;\;\;\;\;\;\;\;\;\;\;\;\;\;\;\;\;\;\;\;\;\;\;\;\;\;\;\;\;\;\;\;\;\;\;\;\;\;\;\;\;\;\;\;\;\;\;\;\;\;\;\;\;\;\;\;\;\;\;\;\;\;\;\;\;\;\;\;\;\;\;\;\;\;\;\;\;\;\;\;\;\;\;\;\;\;\;\;\;\;\;\;\;\;\;\;\;\;$$

Using the results obtained by Equations (36) and (37) in Equation (35), we find:

(38) $${\mathrm{EIRR}}_{F}=\mathrm{Cte}\times {\mathrm{EIRR}}^{0.27808}\times I^{1.2364}$$

To make the obtained Equation (38) more dynamic, the constant (Cte) is defined in terms of IAR data, which is given by Equation (42). The Cte must be obtained by each watershed to take into consideration the variability of each climate area. For this, we propose in Equation (39) the hypothesis that the predicted and real data of the Ol’dekop residuals (IARR’) are almost equal.

(39) $${\;\mathrm{EIRR}}_{F}≅{\mathrm{IARR}}^{\prime }$$

We replace EIRR_F_ in Equation (39) by the formula defined in Equation (38), by doing so, we obtain

(40) $${\mathrm{IARR}}^{\prime }≅\mathrm{Cte}\times {\mathrm{EIRR}}^{0.27808}\times I^{1.2364}\;$$

$$\mathrm{Cte}≅\frac{\mathrm{IARR}\prime }{{\mathrm{EIRR}}^{0.27808}\times I^{1.2364}}$$

**Figure 7:**
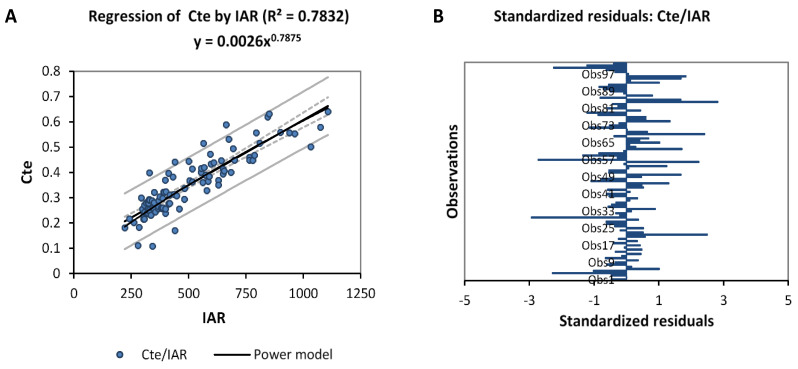
Nonlinear regression graph (**A**), followed by standardized residual analysis histogram (**B**) between inter-annual rainfall (IAR) and Cte data. Real Inter-annual rainfall-runoff residuals (IARR), estimated inter-annual rainfall-runoff residuals by ANN_1_ (EIRR).

Figure 7 shows that Cte data has a very good correlation with IAR, where R^2^ equals 0.7235. When we use the trend equation obtained from the regression model to represent the Cte variable as a function of IAR, we obtain:

(41) $$\mathrm{Cte}=0.0026\times {\mathrm{IAR}}^{0.7875}$$

When we use Equations (22) and (39) in Equation (36), we find:

(42) $${\;\mathrm{EIRR}}_{F}=0.0026\times {\mathrm{IAR}}^{0.7875}\times {\mathrm{EIRR}}^{0.27808}\times I^{1.2364}\;\;\;\;\;\;\;\;\;\;\;\;\;\;\;\;\;\;\;\;\;\;\;\;\;\;\;\;\;\;\;\;\;\;\;\;\;\;\;\;\;\;\;\;\;\;\;\;\;\;\;\;\;\;\;\;\;\;\;\;\;\;\;\;\;\;\;\;\;\;\;\;\;\;\;=0.0026\times {\mathrm{IAR}}^{0.7875}\times {\left(0.01{\times \mathrm{IAR}}^{1.5852}\times S^{-0.1486}\times {\mathrm{Wc}}^{-0.27455}\right)}^{0.27808}\times I^{1.2364}\;\;\;\;\;\;=0.00072\times {\mathrm{IAR}}^{1.2283}\times {\mathrm{Wc}}^{-0.07635}\times S^{-0.04132}\times I^{1.23640}$$

In Equation (43), we used Equations (8) and (42) to define the new form of the Ol’Dekope model used for the IARR estimation.

(43) $${\;\mathrm{IARR}}_{E}=\mathrm{IAR}-\mathrm{IAE}a+{\mathrm{EIRR}}_{F\;}\;=\mathrm{IAR}-\mathrm{ETP}\times \tanh\left(\frac{\mathrm{IAR}}{\mathrm{ETP}}\right)+\left(0.00072\times {\mathrm{IAR}}^{1.2283}\times {\mathrm{Wc}}^{-0.07635}\times S^{-0.04132}\times I^{1.23640}\right)\;$$

## 4. Discussion

**Figure 8:**
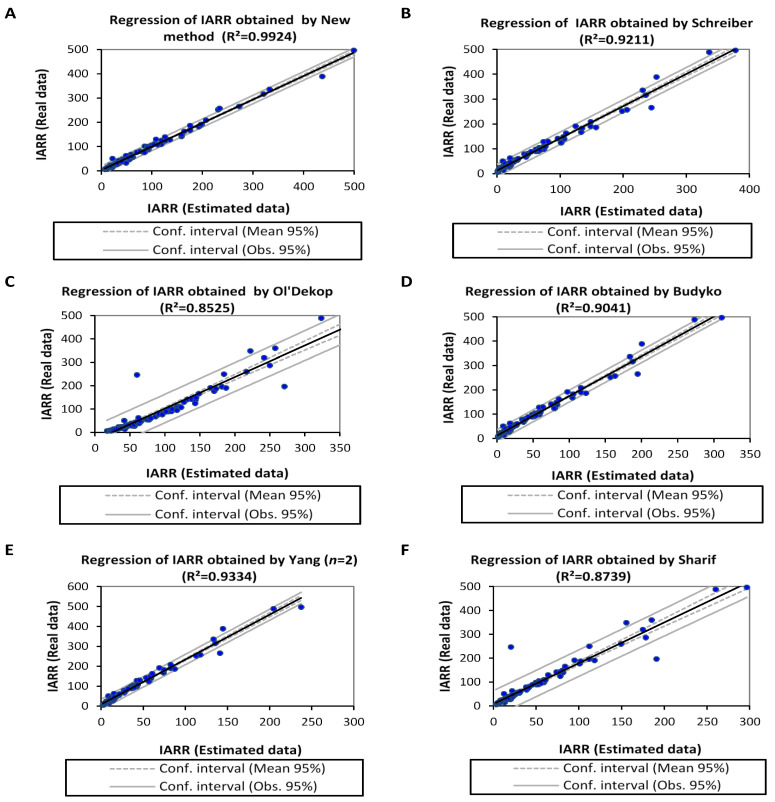
Linear regression graphs between real and estimated inter-annual rainfall-runoff obtained by applying new method (**A**), Schreiber (**B**), Ol’Dekop (**C**), Budyko (**D**), Yang (**E**), and Sharif (**F**) models. Coefficient of determination (R^2^).

Figure 8 shows regression graphs obtained from real and predicted IARR data that are estimated by a set of non-parametric and parametric water balance models most cited in the literature review. These are Schreiber, Ol’Dekop, Budyko, Yang (*n* = 2), and Sharif, as given by Figure 8B–F, respectively. These models were applied to the dataset series used in this modeling to compare the results with the predicted IARR values obtained by the new model, given in Figure 8A. The results demonstrate that the best IARR estimation are obtained by the proposed model, which proved a very good match between the estimated and the actual data (Figure 8A), where the model proved no trend and a very good regression, given by an R^2^, which equals 0.9924. On the other hand, Schreiber and Yang’s models give similar results in which R^2^ equals 0.9211 and 0.9334, respectively (Figure 8B,E). Budyko’s model also gives a good result, and performs less than the previous models, given by an R^2^ equal to 0.9041. On the other hand, Ol’Dekop and Sharif’s models proved a similar performance given by an R^2^ equal to 0.8525 and 0.8739, respectively.

**Figure 9:**
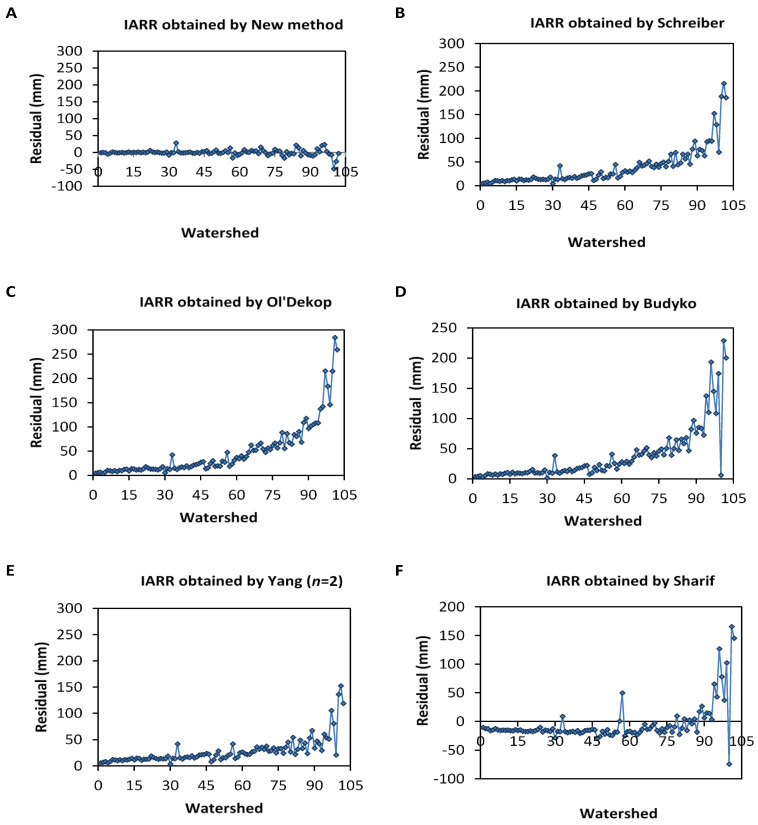
Residual analysis curves between real and estimated interannual rainfall-runoff (IARR), which is obtained by applying a new method (**A**), Schreiber (**B**), Ol’Dekop (**C**), Budyko (**D**), Yang (**E**), and Sharif (**F**) models.

Moreover, in Figure 8C,F, the predicted data obtained by both models show a high tendency. Trend analysis of the new equation compared to the models cited above is given in Figure 9 and Table 7, whereby the figure represents residual curves obtained between measured and predicted data, and the table shows a set of statistical parameters used to analyze the variability and the performance of each model. Figure 9 shows that the new proposition gives the lowest error and no trend of residuals was obtained in Figure 9A when compared to data processed by all runoff models in the 102 sub-basin. Furthermore, the parametric model given by Yang (*n* = 2) is also reliable and can be taken as the second choice to estimate IARR in the region that has a great climatic diversity—i.e., the northern Algeria area.

On the other hand, Sharif’s model shows good results compared to Schreiber, Ol’dekop, and Budyko, where the mean residuals obtained by this last one are symmetrical, distributed between negative and positive value ranges for the axis (X = 0). In this study, the Ol’Dekop model shows a very big tendency for residual data compared to other models, reaching up to 300 mm. In addition, Schreiber and Budyko produce a significant trend of data compared to real observations in watersheds, ranked from 61 to 102, which are located in semi-humid, humid, and hyper-humid climate regions (Figure 3). Table 7 shows that the IARR data series, which is obtained by the proposed model has a similar variability to the real data, given by a coefficient of variation, which equals 9292.465 and 9797.2623, respectively. Moreover, the mean values of both series are 81.4977 and 81.1249, respectively.

**Figure 10:**
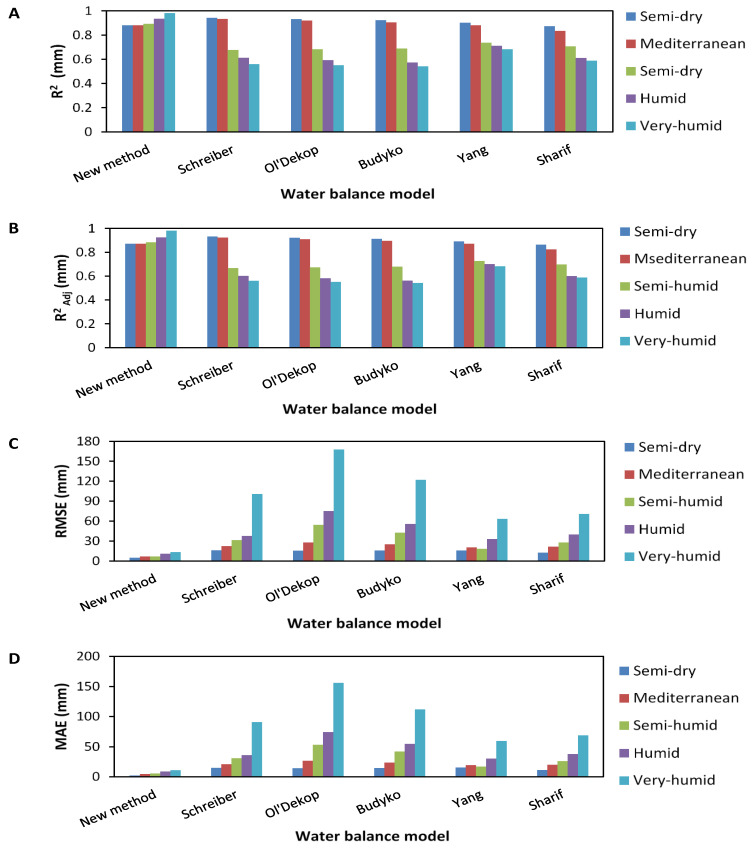
Histograms explaining performance analysis of the new model against a set of parametric (Yang, and Sharif) and non-parametric (Schreiber, Ol’Dekop, and Budyko) water balance models on five climatic regions of Northern Algeria. These were shown by (**A**) determination coefficient (R^2^); (**B**) adjusted coefficient of determination (R^2^_Adj_); (**C**) root mean square error (RMSE); (**D**) mean absolute error (MAE).

The results prove that the variability of data obtained by Schreiber’s model is closer to real data compared to the data obtained by other models, where the coefficient of variation given by this method equals 5772.4952. On the other hand, the statistical analysis of data distribution shows that Yang’s model provides data closer to the real dataset compared to data obtained by Schreiber’s model; whereby the first quartile, the median, the mean, and the third quartile results equal 31.2638, 46.9607, 116.9946, and 86.4257, respectively (Table 7). In this analysis, the new model shows the best performance compared to all models used in this study proven by R^2^, R^2^_Adj_, RMSE, and MAE parameters, which equal 0.9924, 0.9923, 8.5073, and 5.2053, respectively. Figure 10 shows a set of performance tests applied on data series obtained by all models used in this study in five climate regions of northern Algeria.

The results show that the new model has the best reliability and more efficiency in estimating IARR in all watersheds. Where R^2^ and R^2^_Adj_ show values between 0.9 and 0.99, which was proved to be the best performance in the five climatic regions. Furthermore, the model’s performance increases according to the rainfall condition of the basin, leading it to perform more in humid regions than in the arid. The new model is even able to assess flows produced by little precipitation, which is proven in the figure by an R^2^_Adj_ greater than 0.9 in the semi-arid region. On the contrary, the other models are regional and they can be reliable in more arid areas than in humid areas. The R^2^ and the R^2^_Adj_ show that Yang (*n* = 2) and Sharif models perform better than non-parametric models, such as Schreiber, Ol’Dekop, and Budyko models in the semi-humid, humid, and very humid areas. Contrariwise, the performance of these models is better than the Yang and Sharif models in the arid region. The histogram of RMSE and MAE shows that the proposed model has the lowest amount of errors, which are closer to the axis (X = 0) during all observations. However, the other models have more computational residuals, which increase more in humid areas. Ol’Dekop’s model has the highest values of RMSE and MAE observed in semi-humid, humid, and very humid climate areas. The results deduced by this analysis show the power of the proposed model to estimate runoff in different regions of the world. The use of the S and Wc input variables and the calibration applied to the model parameters using the De Martonne series is proof of the dynamicity of the new model and its ability to work with the varying climatic and geomorphological conditions of the watershed, even if it is ungauged. This advantage was justified by the steady performance and lack of a significant trend residual estimation during all the processes carried out in the 102 sub-basins.

## 5. Conclusions and Future Work

This research work aims to propose a new equation derived from Ol’Dekop’s model to estimate the inter-annual rainfall runoff in a large region, which is characterized by climatic diversity. The proposed model was obtained by applying new ANNs to estimate the residual values (IARR’) given when comparing actual and predicted runoff assessed by Ol’Dekop. In this regard, multiple regressions were used as transfer functions and applied to a set of climatic and geomorphological input variables such as IAR, Wc, S, and I. As the first step, the sub-model, namely ANN_1_, proposed to estimate IARR’ in the entire region. Then an improvement was made by classifying output data in each climatic region using the De Martonne index (I), which was also used to calibrate the coefficients of the final model (IRR_F_). The performance analysis shows that the use of the IRR sub equation which is obtained by ANN_1_ as a function of IAR, S, and Wc proved good reliability (R^2^_Adj_ = 0.9518) with a significant error (RMSE = 208.539 mm) when estimating IARR’ in the entire region. Contrariwise, the IRR_F_ sub equation given by ANN_2_ shows an improvement in the estimation model performance, where the R^2^_Adj_ and RMSE equal 0.9789 and 62.5948 mm, respectively. The new equation proposed in this model shows the best performance compared to all parametric and non-parametric water balance models used in this study. This performance is given by an R^2^_Adj_ equal to 0.9923 obtained for the entire northern region of Algeria, with the absence of significant errors proven by residual analyses. Inversely, the compared models are less performed. They are set by the R^2^_Adj_ test between 0.8525 and 0.9333, with a tendency of residuals observed in some watersheds that are located in humid and very humid regions. The comparative performance analysis, which was applied in five climatic regions, shows the best and steady performance of the proposed model in all areas, given by an R^2^_Adj_ more than 0.92, with RMSEs less than 15 mm. However, the other models are regional and perform better in arid regions than in humid. A large trend of residuals is observed by non-parametric models such as Schreiber, Ol’Dekop, and Budyko in the very humid region, where the RMSEs are greater than 100 mm.

Our future work aims to further improve this model designed to estimate rainfall-runoff data in ungauged watersheds using different time scales. We also want to propose a new model using machine learning for estimating rainfall-runoff in sandy basins.

## Figures and Tables

**Table 1:** Statistical criteria used to evaluate the performance of sub-models proposed in all climatic regions.

Criteria	Statistical Formula	Reference
Coefficient of determination (R^2^)	$R^{2}=\frac{\sum_{i=1}^{N}\left(\mathrm{Qoi}-\bar{\mathrm{Qo}}\right)\times \left(\mathrm{Qsi}-\bar{\mathrm{Qs}}\right)}{{\left[\sum_{i=1}^{N}{\left(\mathrm{Qoi}-\bar{\mathrm{Qo}}\right)}^{2}\right]}^{0.5}{\left[\sum_{i=1}^{N}{\left(\mathrm{Qsi}-\bar{\mathrm{Qs}}\right)}^{2}\right]}^{0.5}}$	[39]
Adjusted coefficient of determination (R^2^_Adj_)	$R_{\mathrm{Adj}}^{2}=1-\frac{\left(1-R^{2}\right)\times \left(N-1\right)}{N-K-1}$	[40]
Mean squared error (MSE)	$\mathrm{MSE}=\frac{1}{N}\overset{N}{\underset{i=1}{\sum }}{\left(\mathrm{Qsi}-\mathrm{Qoi}\right)}^{2}$	[41]
Root mean square error (RMSE)	$\mathrm{RMSE}=\sqrt{\frac{\sum_{i=1}^{N}{\left(Q_{\mathrm{si}}-Q_{\mathrm{oi}}\right)}^{2}}{N}}$	[42]
Mean absolute error (MAE)	$\mathrm{MAE}=\frac{\sum_{i=1}^{N}\left|\mathrm{Qoi}-\mathrm{Qsi}\right|}{N}$	[43]
Durbin–Watson coefficient (DW)	$\mathrm{DW}=\frac{\sum_{i=2}^{N}{\left(e_{i}-e_{i-1}\right)}^{2}}{\sum_{i=1}^{N}e_{i}^{2}}$	[44]

**Table 2:** Statistical description and data distribution analysis of a hydro-morphological dataset, obtained between 1967 and 2020 in northern Algeria.

Statistic	IARR’	IAR	IAT	S	WC
No. of data	102	102	102	102	102
Minimum	4.5687	222.0000	11.5833	16.0000	7.1000
Maximum	284.3221	1107.0000	21.7583	4050.0000	179.8000
1st Quartile	13.6200	332.7500	14.2313	194.5000	29.0000
Median	25.9667	415.5000	15.2792	475.0000	45.6000
3rd Quartile	64.3929	607.0000	16.6542	1028.2500	65.5250
Mean	49.1013	494.6529	15.4569	719.7106	51.4108
Variation coefficient	1.1039	0.4044	0.1180	1.0379	0.6313
Standard deviation (*n*)	54.2053	200.0620	1.8236	746.9927	32.4534
*p*-value (Exponential)	0.1353	<0.0001	<0.0001	0.8061	0.0001
*p*-value (Gamma 2)	0.0201	0.0885	0.6043	0.8475	0.9799
*p*-value (GEV)	<0.0001	<0.0001	0.8764	<0.0001	0.7764
*p*-value (Log-normal)	0.1580	0.0805	0.6105	0.7688	0.6973
*p*-value (Logistic)	0.0003	0.0615	0.4604	0.0028	0.3787
*p*-value (Normal)	0.0003	0.0061	0.4358	0.0012	0.0580
*p*-value (Weibull 2)	0.0776	0.0283	0.1929	0.7994	0.6725
*p*-value (Weibull 3)	0.9789	0.8572	0.6136	0.8363	0.8263

Inter-annual rainfall-runoff residuals (IARR’), Inter-annual rainfall (IAR), inter-annual temperature (IAT), Watershed area (S), Watercourse (WC).

**Table 3:** Matrix of correlation between inter-annual rainfall-runoff residual data of Ol’Dkop model and hydro-morphological variables selected for the ANN_S_ modeling.

Variable	IARR’*	S*	WC*	IAT*	IAR*	(IARR’*/S*)	(IARR’*/WC*)	I*
IARR’*	1	0.5141	0.5023	0.0606	0.8513	0.7322	0.8124	0.8052
S*	0.5141	1	0.8929	0.0092	0.1428	0.7923	0.5758	0.1326
WC*	0.4923	0.8929	1	0.0059	0.1266	0.6878	0.6168	0.1180
IAT*	0.0606	0.0092	0.0059	1	0.0804	0.0445	0.0552	0.0126
IAR*	0.8513	0.1428	0.1266	0.0804	1	0.5539	0.6876	0.9505
(IARR’/S)*	0.7322	0.7923	0.6878	0.0445	0.5539	1	0.9267	0.5320
(IARR’/WC)*	0.8124	0.5758	0.6168	0.0552	0.6876	0.9267	1	0.6599
I*	0.8052	0.1326	0.1180	0.0126	0.9505	0.5320	0.6599	1

Inter-annual rainfall-runoff residual data of Ol’Dekop (IARR’), inter-annual rainfall (IAR), inter-annual temperature (IAT), watershed area (S), watercourse (WC), De martone index (I), * input variable linearized by the Ln function, as follow: X_i_* = Ln X_i_.

**Table 4:** Statistical parameters and performance tests of multiple regression models, obtained by ANN_1_.

Statistic	A ^1^	B ^1^	C ^1^	D ^1^	E ^2^
Y	IARR’*	IARR’*	IARR’*	IARR’*	IARR’
X_1_	IAR*	IAR*	IAR*	IAR*	IAR
X_2_	(IARR’/S)*	(IARR’/WC)*	S*	WC*	S
X_3_	-	-	-	-	WC
Coef (a_1_)	1.7166	1.4538	1.7166	1.4538	1.5852
Coef (a_2_)	0.2057	0.3296	−0.2972	−0.5491	−0.1486
Coef (a_3_)	-	-	-	-	−0.2746
Intercept (B)	−6.5738	−5.3946	−5.3201	−3.4494	0.01
No. of observation	102	102	102	102	102
R^2^	0.9301	0.9405	0.9036	0.9105	0.9518
Adjusted R^2^	0.9286	0.9393	0.9006	0.9096	0.9508
MSE	0.0709	0.0603	0.0947	0.0802	208.539
RMSE	0.2663	0.2455	0.3077	0.2833	14.4409
DW	1.7510	1.8557	1.4947	1.4822	0.7094

* input variable linearized by the Ln function, as follow: X_i_* = Ln X_i_, ^1^ linear model ((a_1_X_1_ + a_2_X_2_ + B)), ^2^ final equation represented by power model (B × X_1_a_1_ × X_2_a_2_), no data (-), panels represented in Figure 1A–E, output response (Y), input variable (X), determination coefficient (R^2^), adjusted coefficient of determination (R^2^_Adj_), mean squared error (MSE), root mean square error (RMSE), Durbin–Watson coefficient (DW).

**Table 5:** Statistical parameters and performance tests of multiple regression models, obtained by ANN_2_ for each bioclimatic floor in northern Algeria.

Statistic	Semi-Dry	Mediterranean	Semi-Humid	Humid	Very Humid
Y ^1^	(IARR’SD) *	(IARR’ME) *	(IARR’SH) *	(IARR’H) *	(IARR’VH) *
X_1_	(EIRRSD) *	(EIRRME) *	(EIRRSH) *	(EIRRH) *	(EIRRVH) *
X_2_	(ISD) *	(IME) *	(ISH) *	(IH) *	(IVH) *
Coef (a_1_)	0.50033	0.42496	0.29175	−0.16179	0.28185
Coef (a_2_)	1.16331	1.17641	1.00656	1.34092	1.50295
Intercept (B)	−1.74497	−1.41471	−0.2404	0.45666	−1.60261
No. of observations	45	16	15	11	15
R^2^	0.6501	0.7804	0.6820	0.6533	0.9072
Adjusted R^2^	0.6420	0.7647	0.6675	0.6448	0.9001
MSE	0.0783	0.0296	0.0154	0.0138	0.0173
RMSE	0.2798	0.1721	0.1239	0.1175	0.1314
DW	1.6870	1.7861	1.6803	0.8625	0.7707

^1^ Lineaire model (a_1_X_1_ + a_2_X_2_ + B), * input variable linearized by the Ln function, as follow: X_i_* = Ln X_i_, Semi-Dry (SD), Mediterranean (ME), Semi-Humid (SH), Humid (H), Very Humid (VH), output response (Y), input variable (X), determination coefficient (R^2^), adjusted coefficient of determination (R^2^_Adj_), mean squared error (MSE), root mean square error (RMSE), Durbin–Watson coefficient (DW).

**Table 6:** Performance tests of final and regional inter-annual rainfall-runoff residual models applied to northern Algeria climate regions.

Statistic	IRR_3_	IRR_4_	IRR_5_	IRR_6_	IRR_7_	IRR_F_
No. of observations	102	102	102	102	102	102
R^2^	0.9494	0.9538	0.9443	0.8358	0.9616	0.9841
Adjusted R^2^	0.9490	0.9534	0.9438	0.8342	0.9613	0.9789
MSE	151.4110	138.3785	166.6840	492.0888	114.9051	62.5948
RMSE	12.3049	11.7634	12.9106	22.1831	10.7194	5.9117
DW	1.7324	1.6142	1.5675	1.8477	1.4434	0.5250

Semi-Dry (3), Mediterranean (4), Semi-Humid (5), Humid (6), Very Humid (7), final inter-annual rainfall-runoff residuals model (IRR_F_), regional interannual rainfall-runoff residuals model (IRR), determination coefficient (R^2^), adjusted coefficient of determination (R^2^_Adj_), mean squared error (MSE), root mean square error (RMSE), and Durbin–Watson coefficient (DW).

**Table 7:** Statistical tests and performance analysis of estimated inter-annual rainfall-runoff final results (IARR), obtained by applying a new method, Schreiber, Ol’Dekop, Budyko, Yang, and Sharif models.

Statistic	Real Data	New Method	Schreiber	Ol’Dekop	Budyko	Yang (*n* = 2)	Sharif
No. of obs	102	102	102	102	102	102	102
Minimum	7	7.0371	0.5553	2.039	1.2984	17.1006	3.0295
Maximum	497	514.9642	377.8251	237.4534	310.7265	351.4336	296.4772
1st Quartile	20.125	19.394	5.9808	6.7714	6.3305	31.2638	9.9495
Median	39	35.963	18.6804	13.9953	16.4043	46.9607	20.2944
3rd Quartile	104.25	97.8777	72.7747	41.4154	57.3576	116.9946	58.088
Mean	81.4977	81.1249	52.8807	32.3539	42.8715	86.4257	44.2095
Variance (*n*)	9292.465	9798.2623	5472.4952	1805.6423	3450.3124	4759.8256	3013.5653
Std. Dev (*n*)	96.3974	98.9862	73.9763	42.4929	58.7394	68.9915	54.8959
Var. Coef	1.1828	1.2202	1.3134	1.3701	1.3989	1.2417	0.7983
R^2^	1	0.9924	0.9211	0.8525	0.9041	0.9334	0.8739
R^2^_Adj_	1	0.9923	0.9203	0.8524	0.9074	0.9333	0.8729
RMSE	0	8.5073	12.865	30.6084	20.739	12.2717	12.5336
MAE	0	5.2053	7.8716	18.7281	12.689	7.5089	7.6688
Mean A. Dev	67.8752	68.5061	29.2838	53.3262	38.8325	41.5967	53.2656
Std. E. Mean	9.5919	9.8495	4.2282	6.8649	5.4624	5.8448	7.3609

Standard deviation (Std. Dev), variation coefficient (Var. Coef), determination coefficient (R^2^), adjusted coefficient of determination (R^2^_Adj_), root mean square error (RMSE), mean absolute error (MAE), mean absolute deviation (Mean. A. Dev), standard error of the mean (Std. E. Mean).

## Data Availability

The data presented in this study are available on request from the corresponding author.

## Abbreviations

IAR	Inter annual rainfall
IAT	Inter annual temperature
S	Watershed area
WC	Watercourse
I	De Martonne index
IARR	Inter annual rainfall runoff
IAE_0_	Inter annual potential evapotranspiration
IAE_a_	Inter annual actual evapotranspiration
IARR_R_	Real inter annual rainfall runoff
IARR_E_	Inter annual rainfall runoff estimated by Ol’Dekop model
IARR’	Inter annual rainfall-runoff residual data obtained from Ol’Dekop model
(X_i_)*	Linearized variable
EIRR	Inter annual rainfall runoff residual data estimated by ANN_1_
EIRR_F_	Inter annual rainfall runoff residual estimated by ANN_2_
IRR	ANN transfer function given by regression equation
ANN	Artificial neuron network
ANN_1_	Basic artificial neuron network model
ANN_2_	Dynamic artificial neuron network model
SD	Semi-dry
ME	Mediterranean
SH	Semi-humid
H	Humid
VH	Very humid
R^2^	Coefficient of determination
R^2^_Adj_	Adjusted coefficient of determination
MSE	Mean squared error
RMSE	Root mean square error
MAE	Mean absolute error
DW	Durbin–Watson coefficient
